# Research on the Impact of Customer Participation in Virtual Community on Service Innovation Performance— The Role of Knowledge Transfer

**DOI:** 10.3389/fpsyg.2022.847713

**Published:** 2022-03-23

**Authors:** Jianhua Wang

**Affiliations:** ^1^Business School, Changshu Institute of Technology, Suzhou, China; ^2^Evergrande School of Management, Wuhan University of Science and Technology, Wuhan, China

**Keywords:** virtual community, customer participation, service innovation, knowledge transfer, mediation effect

## Abstract

Internet technology has given birth to continuous changes in business model and format innovation. With increasingly critical consumers, blowout development model and format innovation, enterprises are increasingly aware of the importance of customer participation in service innovation. At the same time, the development of information technology provides convenient conditions for communication between enterprises and customers, and online virtual community also provides a platform for customers to participate in the process of enterprise service innovation in an instant. Based on the theory of customer participation, knowledge transfer and service innovation performance, this paper explores the influence mechanism of customer participation in virtual community on service innovation performance, and analyzes the mediating role of knowledge transfer. Through the analysis of the results of the questionnaire, the relevant hypotheses are verified. The results show that customer participation in virtual community has a positive impact on service innovation performance. Customer participation helps enterprises obtain relevant knowledge such as customer needs and reduce barriers to knowledge sharing. In addition, enterprises will acquire customer knowledge about new products, which provides the possibility for the development of new products and services, thereby enhancing the enterprises’ service innovation performance. Knowledge transfer plays a part of mediating role between customer participation and service innovation performance. In the process of enterprises’ service innovation, customers mainly participate in the enterprise by means of knowledge transfer and help the enterprise improve service innovation performance.

## Introduction

With the continuous update and development of Internet technology, business model and format innovation continue to emerge, which brings huge opportunities and challenges to enterprises. With fierce market competition, diversified and customized consumers, blowout development model and format innovation, enterprises can only gain a foothold in the market by continuously carrying out service innovation. Enterprises and researchers are becoming more and more aware of the importance of customer participation, believing that customers and enterprises can realize value co-creation together, and making full use of customer resources for service innovation is the source of the core competitiveness of enterprises. Value co-creation theory believes that customers and enterprises create value together, and customer participation becomes a key factor in determining the success or failure of innovation (Vezzoli, 2014). Customer participation has become an important factor for enterprises to break through organizational boundaries, integrate external resources, and achieve open innovation. The importance of customer participation for product or service innovation performance has been widely recognized (Mourtzis et al., 2015; Sakao and Lindahl, 2015). Many enterprises face pressure from customer interactions in service innovation projects and obtain input from them. Customer participation can serve as a viable way of dealing with customer pressure and promoting product innovation (Chen and Liu, 2020). Therefore, in order to learn from customers and then discover and meet customers’ potential needs, we should attach importance to customer participation in the service innovation process and explore effective ways to learn from customers in the service innovation process (Alam, 2002). At the same time, the development of information technology also provides a convenient platform for enterprises to communicate with customers. More and more enterprises build online virtual communities such as official forums, official weibos, brand communities, and WeChat groups. Customers can participate in the process of enterprises’ service innovation through these virtual communities, and enterprises can make full use of customers’ relevant knowledge. In the Internet environment, customer participation in service innovation is a two-way coupling innovation, and it is also a process of value co-creation of virtualization and socialization. With a large number of participants, the depth and width of participation has greatly expanded, and the contribution has become larger and larger. More and more enterprises have shifted their focus to value co-creation activities between customers and enterprises, fully mobilizing the wisdom of customer groups, and providing conditions for them to create new personalized services. For example, Bank of America (new banking service function test), Starbucks (my Starbucks idea), McDonald’s (fries creative eating contest) have explored on this road, and established mechanisms and means for customer and enterprise interaction and cooperative innovation, such as innovation markets, customer forums, and crowdsourcing platforms.

Although enterprises have realized the importance of customer participation, they still do not pay enough attention to customer participation. Current literature research found that customer participation was conducive to the improvement of innovation performance. Gustafsson et al. (2012) found that customer participation was conducive to improving product innovation and market share. In addition, many studies have shown that customer participation played an important role in improving corporate financial performance and product innovation performance. However, “customer participation” only pays attention to the general status of customer participation, and does not reflect the interactive process. In particular, research on virtual communities and new interactive environment inside and outside enterprises is relatively lacking. Compared with the existing literature, the main contributions of this paper are as follows: First, it constructs a theoretical model of the relationship between customer participation and service innovation performance and conducts an empirical analysis to answer the key factors such as the internal mechanism of customer participation in improving service innovation performance. Second, it emphasizes the interactive nature of customer participation, and places customer participation in the context of virtual communities and new types of interactions inside and outside the enterprises. Third, based on the perspective of knowledge transfer, it explores the mediating role of knowledge transfer in customer participation and service innovation performance, and answers questions such as the boundary conditions of customer participation on improving service innovation performance. In fact, one of the important reasons for customer participation to improve service innovation performance is the transfer and sharing of customer knowledge.

The paper is organized as follows: The following two sections present the relevant literature review and hypotheses development. Research design is outlined, followed by data analysis and results. Conclusion, implications, as well as the limitations and future research are provided to conclude this paper.

## Literature Review

### Customer Participation in Virtual Community and Service Innovation Performance

In the context of the continuous development of Internet technology, it is becoming more and more important for enterprises to interact with customers in real time through platforms such as virtual communities. Rheingold (1993) first proposed the concept of virtual community, which was defined as “a group of people who communicate with each other through a computer network, share various knowledge and information, and form a personal network of relationships.” Preece (2001) and Plant (2004) believed that based on common interests, goals, etc., the network “members” in virtual communities could gather to communicate without time and space restrictions, therefore virtual communities were social communities for people to communicate online around certain interests and needs. Jones and Rafaeli (2000) defined virtual community as a community formed on the Internet based on network technology and software technology, through close contact between people. Hunter (2002) believed that members of virtual communities not only participated in interaction, but also learnt from each other, and proved that knowledge and information resources in the group were closely related to shared interests. This shows that virtual community members need to be contributors, not just viewers or consumers. Dholakia et al. (2004) believed that virtual community members would contribute knowledge through the virtual community, bringing new needs and choice information, new experience, and new product awareness information to the enterprise. The members of the virtual community have contributions to the transfer of social knowledge and enterprise innovation. Therefore, the virtual community is mainly based on a certain common demand or interest, through the network environment to establish a multi-party participation, continuous communication information exchange and sharing platform.

Customer participation emerged in the field of service marketing. With the continuous evolution of market relations and the continuous integration of products and services, customer participation has further expanded to various fields such as brand co-creation, product customization design, and product innovation. Customer participation involves multiple processes of the enterprise, and pays more attention to the communication and interaction behaviors between customers and innovative product developers in the process of participating in enterprises’ innovation, mainly participating in the process of new product development (Claycomb et al., 2001; Cragin, 2003). Divided from specific dimensions, customer participation includes multi-dimensional variables including information sharing, responsible behavior and personal interaction (Ennew and Binks, 1999). Alam (2002) believed that customer participation in service innovation includes four elements: “customer participation goal,” “customer participation stage,” “customer participation intensity” and “customer participation model,” and put forward measurement questions, which had been widely accepted by scholars. Carbonell et al. (2010) believed that customer participation was measured through four items: the frequency of meeting with customers; the degree of consultation with customers; the quantity of the project customer participated; and the number of customer participation tools used. Cui and Wu (2016) divided them into three forms according to the degree of customer participation in new product innovation: information resource providers, co-developers, and independent innovators. Due to the strong interaction and communication in the process of customer participation in virtual communities, this paper divides customer participation into three dimensions: information sharing, cooperative behavior, and interpersonal interaction.

The concept of innovation performance was derived from “technical efficiency,” but it was Amabile (1996) that clearly put forward the theory of innovation performance. He believed that innovation performance was that when employees encounter new problems at work, they could find new ways to solve the problems. Janssen and Van Yperen (2004) clearly put forward the concept of enterprises’ innovation performance based on the personal performance structure model. He defined new ideas that are generated by employees in the process of actively completing or promoting personal work as enterprises’ innovation performance. The Organization for Economic Co-operation and Development [OECD] (2005) believed that innovation performance was composed of multiple forms, including the effective replacement of products or services, optimization in the production or service process, the economic benefits of using different marketing methods, or the commercial profits created by new organizational activities. Scholars mainly used single-dimensional and multi-dimensional forms to describe innovation performance. Hagedoom and Cloodt (2003) believed that innovation performance was mainly divided into four categories of indicators, namely innovation cost, number of patents declared, number of patents owned, and number of new product developments. Beldersbos et al. (2004) believed that the measurement of innovation performance should be divided into two aspects: product and process innovation. Ellonen et al. (2009) determined that enterprises’ innovation performance was related to the internal and external levels after many investigations, specifically the integration of internal resources and the grasp of external opportunities. Jia et al. (2016) believed that it was currently in the initial stage of product and service innovation performance evaluation, and it was appropriate to use simplified indicators to measure product and service innovation performance. This paper defines service innovation performance as the ability and degree of an enterprise to develop new products and services or improve existing products and services in order to meet the needs of customers and itself, so as to maintain the enterprise’s competitive advantage. Based on the perspective of service innovation efficiency and effectiveness, this paper uses service innovation process performance and service innovation result performance to define service innovation performance.

With the continuous development of Internet technology and the continuous emergence of innovations in business models and formats, scholars are increasingly aware of the importance of customer participation in improving the performance of enterprises’ service innovation. Scholars believe that customer participation can promote the optimization of enterprises’ service quality and also promote the innovation and development of enterprises, especially service-oriented enterprises. By systematically combing the evolutionary context of customer participation, it can be seen that customer participation in the service field focuses on the study of individual customers involved in the service provision process under the B2C model. Gruner and Homburg (2000) showed that customer participation in a specific stage of new product development had a positive impact on the success of new product development. Kristensson et al. (2002) believed that enhancing the interaction between customers and producers would improve the level of creativity. Therefore, the process of customer participation could be an additional contribution to the product development. Carbonell et al. (2010) showed that customer participation had a positive impact on technology quality and innovation speed, and an indirect impact on competitive advantage and sales performance. Fang et al. (2008) found the relationship between the depth and width of customer participation and innovation performance through empirical research. Customer participation in the field of product manufacturing focuses on the research of co-production such as customer information provision, contact strength, design participation, etc (Mustak et al., 2013). In particular, customer participation in new product development has received widespread attention. Scholars represented by Bonner (2010) and Morgan et al. (2018) had discovered that customer participation had a positive impact on the performance of new product development and innovation. Zine et al. (2014) and Sjudin et al. (2016) showed that customers and enterprises were the co-creators of the value of the product service system, which indirectly illustrated the importance of customer participation in the value creation of the product service system. Hsieh et al. (2004) believed that customer participation in the service innovation process of the enterprise in the form of time, effort and information sharing. Customers clearly told the enterprise their information and service needs, and at the same time served as opinion providers. “Interactive learning” behavior occurred between enterprises and customers (Strambach, 2001). This behavior could effectively reduce the cognitive burden and reduce enterprises’ information redundancy (Zhong et al., 2012), which was conducive to the acquisition and reorganization of enterprises’ innovation knowledge, and was conducive to the generation of service innovation results (Orfila-Sintes and Mattsson, 2009). Ennew and Binks (1999) and Fang (2008) believed that customers acted as co-developers and their participation behavior could propose specific ideas or innovative solutions for enterprises’ innovation activities (Arnold et al., 2011). Their cooperative behavior could meet the complex and subtle dynamic needs of customers, reduce innovation costs (Yoo et al., 2020), improve the speed of new product innovation and market entry (Rautela et al., 2020), and enable the innovation process to be completed smoothly, which promoted the improvement of innovation process performance (Yalley, 2021). Customer participation in service innovation had a certain degree of interaction (Chesbrough, 2011). This interaction included many interpersonal factors such as trust, reliability, support, and commitment, and was an emotional communication. This kind of communication was conducive to the construction of reciprocity and trust between customers and enterprises (Ennew and Binks, 1999). Moreover, it could reduce the cost of supervision and negotiation (Dyer and Singh, 1998), and sped up the exchange of knowledge and skills (Choi et al., 2010), Thereby, it would finally help enterprises to improve the service process, to improve the effectiveness of service delivery, and enhance the organization’s innovation ability.

### Knowledge Transfer

With the development of Internet technology, society has gradually entered the era of knowledge economy. As a means to effectively expand the scope and quality of knowledge, knowledge transfer has received great attention. Knowledge transfer among enterprises is widely recognized as an important source of competitiveness (Majuri, 2022). Knowledge transfer can help the knowledge-receiving enterprises overcome the internal knowledge resources needed for innovation and meet their diversified knowledge needs, which can also help the knowledge-receiving enterprises obtain heterogeneous knowledge from their partners and complement each other. The differences produced by knowledge integration have created demands for new knowledge and product innovation. Teece (1977) first proposed the concept of knowledge transfer, which was mainly used in the field of innovative research, and then gradually extended to use in different fields. Scholars have roughly two definitions of knowledge transfer: one thinks that knowledge transfer is a process, and the other thinks that knowledge transfer is a result. Szulanski (1996) and Szulanski et al. (2004) proposed that knowledge transfer was the purposeful and planned transmission and reception of knowledge between or within different enterprises, which described the knowledge movement. Griffith et al. (2001) focused on knowledge transfer related to the market environment and corporate practices, and designed a total of 10 measurement items. Cummings and Teng (2003) measured knowledge transfer from three aspects: “the receiver obtains the ownership of the transferred knowledge,” “commitment to the transferred knowledge” and “the degree of satisfaction with the transferred knowledge.” Wijk et al. (2008) defined knowledge transfer as the process of the exchange and acceptance of knowledge between enterprises. In the process of knowledge transfer, it was not only necessary to provide and receive knowledge, but also to integrate the received knowledge. Sarala et al. (2016) pointed out that knowledge transfer was the process of successfully transferring tacit, encoded and complex knowledge resources between enterprises. Knowledge transfer could be operated by various mechanisms in accordance with knowledge types. Explicit knowledge was relatively easy to transfer beyond organizational boundaries, and tacit knowledge based on experience and learning was difficult and expensive to transfer, requiring rich communication and interaction (Ranft and Lord, 2002). Despite the different types of knowledge resources, transferring both explicit and tacit knowledge was of high value (Hau et al., 2013). Learning from the research of scholars, this paper believes that knowledge transfer is a process in which customers transfer knowledge to the enterprise through a series of interactions, and the enterprise further absorbs and applies the acquired knowledge.

Scholars have conducted in-depth discussions on the influencing factors and mechanisms of knowledge transfer. Numerous studies have demonstrated that structural and capacity-supporting components can facilitate knowledge transfer (Inkpen and Tsang, 2005; Ozkan-Canbolat and Beraha, 2019). Knowledge transfer depends on the presence of an atmosphere that promotes cooperation and trust, as well as actors’ feelings of satisfaction with their efforts (Abdullah et al., 2011). Knowledge transfer is more successful when there are strong interpersonal relationships and reciprocal, cohesive shared norms between customers and enterprises. Chen et al. (2014) believed that mutual trust, frequent communication and effective coordination could improve the effectiveness of knowledge transfer. Duvivier et al. (2019) argued that knowledge transfer not only needed to focus on providers, but also on receivers. However, different types of knowledge were not equally easy to transfer (Lagerström and Andersson, 2003). There were various difficulties in the knowledge transfer process, including the willingness of the provider, the absorptive capacity and willingness of the receiver, and the quality of learning and its causal ambiguity, etc. (Liu et al., 2015). With the introduction of the knowledge-based view of the enterprise, scholars shown that both expatriation (Salleh and Nankervis, 2015) and inpatriation (Harzing et al., 2016) were critical knowledge transfer channels. In practice, enterprises also taken the initiative to manage customer relationships and create social capital to prepare for effective and efficient knowledge transfer (Inkpen and Tsang, 2005). With modern and advanced network technology as the medium, the cooperation between customers and enterprises has become more and more convenient, and the knowledge transfer from customers has a positive impact on the service innovation performance. Enterprises provide convenient conditions for customer participation through platforms such as virtual communities. Customer participation is an important element of enterprises’ service innovation. The form, depth and width of customer participation, and how the enterprises receive customer knowledge are all very important. Customer participation is a prerequisite for customer knowledge transfer. The information sharing, cooperative behavior and interpersonal interaction of customer participation are essentially the process of knowledge transfer. Therefore, customer participation and customer knowledge transfer are interrelated. Customer participation through virtual communities such as purchases, incentivized recommendations, social media conversations about brands, and customer feedback provide opportunities for explicit and tacit knowledge transfer. Discussions and feedback provided by customers about products and services on community platforms can both have knock-on effects on a broad potential group and help organizations improve their products and services, or generate new ideas for new products (Pansari and Kumar, 2017). Some scholars believed that the quality of customer knowledge transfer could be assessed by the frequency and intensity of customer knowledge sharing (Weber and Weber, 2007). Eng et al. (2014) argued that implementation quality and sustainability were key points for knowledge transfer, and strategies with a well-defined knowledge transfer plan were more likely to be successful.

### Hypotheses Development

#### The Impact of Customer Participation on Service Innovation Performance

Customer participation could benefit enterprises directly or indirectly. These benefits could be seen in the form of enterprise performance (Pansari and Kumar, 2017). Harmeling et al. (2017) also found that customer engagement could add value to enterprises, such as network assets, persuasion capital, knowledge and creativity. Customers participate in enterprises’ innovation activities with positive emotions, engage in enterprises’ marketing research extensively, and maintain communication and interaction with enterprises. Therefore, customer participation is helpful to the development of new product development, effectively enhancing enterprises’ R&D and innovation capabilities, and improving enterprises’ innovation performance. In addition, customers are closer to the market and have a better understanding of the market’s information needs and competition. The participation and attention of customers would enable enterprises to accurately allocate resources to innovation (Fang, 2008). Customers, as information providers, co-developers, and interpersonal communicators, contribute their knowledge, experience and skills to innovation activities, which are beneficial to enterprises’ value creation. Therefore, in terms of service innovation, customer participation positively affect enterprises’ service innovation performance (Arnold et al., 2011). According to the three dimensions of customer participation and the two dimensions of service innovation performance, this paper proposes the following hypotheses:

H1: Customer participation has a positive impact on service innovation performance.
H1a: Information sharing of customer participation has a positive impact on service innovation process performance.
H1b: Cooperative behavior of customer participation has a positive impact on service innovation process performance.
H1c: Interpersonal interaction of customer participation has a positive impact on service innovation process performance.
H1d: Information sharing of customer participation has a positive impact on service innovation result performance.
H1e: Cooperative behavior of customer participation has a positive impact on service innovation result performance.
H1f: Interpersonal interaction of customer participation has a positive impact on service innovation result performance.

#### The Mediating Role of Knowledge Transfer

In virtual community, the relevant knowledge provided by customers is very important for enterprises’ service innovation. The stage of innovation project from design to commercialization is essentially a process of knowledge transfer between innovation subjects. The relevant information provided by customers enables enterprises to more accurately grasp market needs, so as to innovate existing products and services, and achieve the purpose of improving enterprises’ innovation performance. Of course, enterprises need to screen and sort the received customer knowledge, and absorb and use useful knowledge, which is effective to knowledge transfer. Therefore, the knowledge transfer between customers and enterprises has a significant impact on the service innovation performance. He (2004) found that in the development of customer participation system, there was a significant relationship between customer knowledge participation and team performance, and knowledge interaction (knowledge acquisition and knowledge development) played an mediating role between customer knowledge participation and team performance. Due to the development of Internet technology, customers are gradually integrated into the dialog with producers. Customers could become a new source of enterprises’ competitiveness through their own knowledge or through learning other customers’ knowledge (Prahalad and Ramaswamy, 2000). Xin et al. (2021) believed that in the digital age, inspired by the theories of knowledge-based and absorptive capacity, customer participation and knowledge management had positive effects on service innovation performance, respectively, and knowledge management played an mediating role in the impact of customer participation on service innovation performance. In addition, some studies have pointed out that the customer toolbox provides a platform for cooperation between enterprises and customers. The customer toolbox was conducive to obtaining program information and innovative ideas in the user field, and transferring customer creative ideas to producers (Piller and Walcher, 2006). Therefore, we can speculate that knowledge transfer plays an mediating role in the relationship between customer participation and enterprises’ innovation performance. Accordingly, this paper puts forward the following hypothesis:

H2: Knowledge transfer plays an mediating role between customer participation and service innovation performance.

**FIGURE 1:**
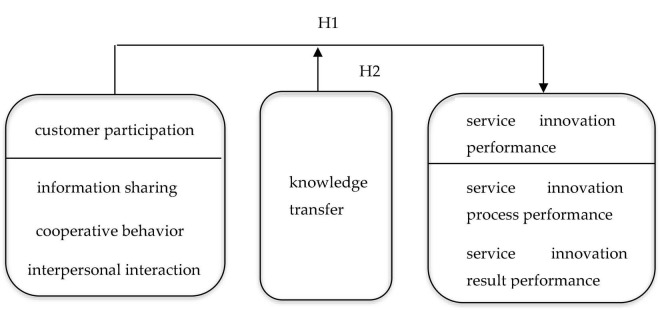
Theoretical model.

In summary, the theoretical model of this paper is shown in Figure 1.

## Research Design

### Research Objects and Questionnaires

In this paper, the questionnaires are designed into two parts: basic personal situation and scale measurement. In the basic personal situation part, the investigation item is set up, that is, it must be an individual with virtual community participation behavior in order to screen out the target subjects. In the measurement part of the scale, the five-level Likert scale is used to measure each item, 1 means completely disagree, and 5 means completely agree. Before questionnaires are formally distributed, this paper first conducts a small-scale interview test with 20 people on the semantic clarity and independence of each measurement item, including experts in the field, as well as a number of students and social workers from different fields. According to the interview and test results, the official questionnaires are obtained by modifying. This paper uses the questionnaire platform to produce formal questionnaires and distribute them on a large scale through various channels such as virtual communities to complete the questionnaires data collection. In this study, a total of 514 questionnaires are collected, and the questionnaires that took less than 1 min to fill in and answered the same questions in the target sample book are excluded. In the end, 492 valid questionnaires are obtained for the target participant, and the effective rate of questionnaires are 95.72%.

### Scale Design

The scales used in this study are mainly derived from mature scales in the academic community, and these scales are proven to have good reliability and validity. The specific measurement of each variable is as follows.

(1) The independent variable is customer participation. According to Ennew and Binks (1999) and Yen et al. (2004) and other scholars on the dimension division and scales of customer participation, this paper determines the measurement of customer participation from three dimensions: information sharing, cooperative behavior and interpersonal interaction.
(2) The dependent variable is service innovation performance. According to the scales used by scholars such as Hsueh and Li (2010) to measure innovation performance, this paper determines the measurement of service innovation performance from two dimensions: service innovation process performance and service innovation result performance.
(3) The mediating variable is knowledge transfer. Combining the definition and measurement scales of knowledge transfer by scholars, this paper adopts the knowledge transfer measurement scales proposed by Griffith et al. (2001).

## Data Analysis and Results

### Reliability and Validity Analysis

AMOS24.0 and SPSS20.0 are used to analyze the reliability and validity of the measurement model (Table 1). The results show that the Alpha value of each variable is greater than 0.8, indicating that the various scales have high internal consistency, and the reliability of the scale has passed the test. The KMO value of each variable is greater than 0.8, and the probability p value of Bartlett’s sphere test is 0.000 (*p* < 0.01).

**TABLE 1:** Reliability and validity analysis of variables.

Variables	Cronbach’s Alpha	KMO
Information sharing	0.909	0.914
Cooperative behavior	0.848	0.882
Interpersonal interaction	0.870	0.863
Knowledge transfer	0.871	0.915
Service innovation process performance	0.887	0.847
Service innovation result performance	0.895	0.861

The validity of the scale is further verified by confirmatory factor analysis (Table 2). The results show that the combined reliability (CR) of each latent variable is greater than 0.7, and the average variance extraction (AVE) is greater than 0.5, indicating that the various fitness indicators of the model are within the range of the reference standard, and it has good fitting validity and convergence validity.

**TABLE 2:** Confirmatory factor analysis results.

Variables		Convergence validity		Fitness index						
		CR	AVE	χ2/df	GFI	RMSEA	RMR	CFI	AGFI	TLI
CP	IS	0.898	0.639	2.042	0.938	0.068	0.026	0.973	0.895	0.961
	CB	0.873	0.586							
	II	0.839	0.642							
KT		0.916	0.685	2.166	0.954	0.069	0.016	0.979	0.917	0.970
SIP	SIPP	0.869	0.625	2.168	0.968	0.069	0.014	0.987	0.923	0.975
	SIRP	0.877	0.640							
Guideline		> 0.7	>0.5	< 3	> 0.9	< 0.08	<0.05	> 0.90	>0.90	> 0.90

*CP = customer participation; IS = information sharing; CB = cooperative behavior; II = interpersonal interaction; KT = knowledge transfer; SIP = service innovation performance; SIPP = service innovation process performance; SIRP = service innovation result performance.*

### Correlation Analysis

The results show that the correlation coefficients between variables are all greater than 0.4, and are significant at the 0.01 level, indicating that there is a significant correlation between the latent variables (Table 3). The AVE square roots of all variables are greater than the correlation coefficients between this variable and all other variables, and the correlation coefficients between all variables are less than 0.8, indicating that the variables have good discrimination validity.

**TABLE 3:** Correlation analysis between variables.

Variables	1	2	3	4	5	6
Information sharing	0.799					
Cooperative behavior	0.683**	0.766				
Interpersonal interaction	0.763**	0.744**	0.801			
Knowledge transfer	0.776**	0.721**	0.797**	0.828		
Service innovation process performance	0.751**	0.745**	0.787**	0.783**	0.791	
Service innovation result performance	0.765**	0.774**	0.791**	0.670**	0.750**	0.800

** is a significant correlation at the 0.01 level (two-sided), the value on the diagonal is the square root of AVE, and the rest are the correlation coefficients between the variables.

### Regression Analysis

According to the correlation analysis, there is a certain correlation between customer participation, knowledge transfer and service innovation performance. In order to further determine the relationship between the variables and their dimensions, this paper uses multiple regression analysis to verify the hypotheses. First, a regression analysis model (model 1) of customer participation dimensions (independent variables) and service innovation process performance (dependent variable) is constructed. Second, the regression analysis model (model 2) of customer participation dimensions (independent variables) and service innovation result performance (dependent variable) is constructed. The results are shown in Table 4.

**TABLE 4:** Regression analysis results.

Regression models			Standardization statistics			Collinearity statistics	Model parameters	
Model	Independent variables	Dependent variable	β	t	Sig	VIF	F	R^2^
1	IS	SIPP	0.394	7.678	0.000	2.532	246.658	0.768
	CB		0.369	7.144	0.000	2.576		
	II		0.203	3.474	0.001	3.292		
2	IS	SIRP	0.308	5.568	0.000	2.532	202.044	0.727
	CB		0.333	5.975	0.000	2.576		
	II		0.301	4.774	0.000	3.292		

*IS = information sharing; CB = cooperative behavior; II = interpersonal interaction; SIPP = service innovation process performance; SIRP = service innovation result performance.*

In Model 1, the regression coefficients of information sharing, cooperative behavior, and interpersonal interaction of customer participation show a significant level of 0.01 (*p* < 0.01), and both F-value and R^2^ meet statistical standards, indicating that the model is very suitable. The VIF value is less than 10, indicating that the collinearity between the variables is very low. H1a, H1b, and H1c pass the test, and these show that each dimension of customer participation will have a positive impact on the service innovation process performance. Among them, the information sharing dimension has the greatest impact (standard coefficient is 0.394), followed by the cooperative behavior dimension (standard coefficient is 0.369), and the interpersonal interaction dimension is the smallest (standard coefficient is 0.203).

In Model 2, the significant level of each dimension of customer participation is 0.000, and the VIF value is less than 10, indicating that the collinearity between the variables is very low. H1d, H1e, and H1f pass the test, and those show that each dimension of customer participation will have a positive impact on the service innovation result performance. Among them, cooperative behavior has the greatest impact (standard coefficient of 0.333), followed by the information sharing (standard coefficient of 0.308), and the interpersonal interaction has the smallest (standard coefficient of 0.301).

In summary, this paper draws the conclusion: customer participation has a significant positive impact on service innovation performance, and hypothesis H1 is supported.

### Analysis on the Mediating Effect of Knowledge Transfer

First, stepwise regression analysis is used to obtain the regression test results (Table 5).

**TABLE 5:** Regression test of the mediating effect of knowledge transfer.

	Equation	Coefficient	Error	p
Step 1: customer participation ⇒ knowledge transfer	m = 0.3828 + 0.9164x	a = 0.9164	SE = 0.0286	0.0000
Step 2: customer participation ⇒ service innovation performance	y = 0.3764 + 0.9207x	c = 0.9	SE = 0.0301	0.0000
Step 3: customer participation, knowledge transfer ⇒ service innovation performance	y = 0.1497 + 0.3782x + 0.5921m	b = 0.5921 c’ = 0.3782	SE = 0.0586 SE = 0.0579	0.0000 0.0000
				

In the first step, the regression analysis with customer participation as the independent variable and knowledge transfer as the dependent variable shows that the regression coefficient of customer participation on knowledge transfer (*a* = 0.9164) is significant (*p* = 0.0000). In the second step, the regression analysis with customer participation as the independent variable and service innovation performance as the dependent variable shows that the regression coefficient of customer participation on service innovation performance (*c* = 0.9207) is significant (*p* = 0.0000). In the third step, the regression analysis is performed with customer participation and knowledge transfer as independent variables and service innovation performance as dependent variables. The results show that the regression coefficient of customer participation on service innovation performance (*c*’ = 0.3782) is significant (*p* = 0.0000), and the regression coefficient of knowledge transfer on service innovation performance (*b* = 0.5921) is significant (*p* = 0.0000). At the same time, it (c’ < c) indicates that knowledge transfer plays a part of the mediating role in the relationship between customer participation and service innovation performance.

Second, in order to further test the mediation effect, this study uses Hayes’ Process program to conduct Bootstrap sampling test, set a significant level of 0.05, and sample 5000 times. The results (Table 6) show that the confidence interval of the mediation effect is (0.3770, 0.6931), and the confidence interval does not contain 0, so the mediation effect is significant. Therefore, knowledge transfer plays an mediating effect in customer participation and service innovation performance. Hypothesis H2 is supported.

**TABLE 6:** Bootstrap analysis of mediation effect.

	Item	Effect	SE	LLCI (Lower limit)	ULCI (Upper limit)	P
Direct effect	CP⇒SIP	0.3782	0.0586	0.2626	0.4937	0.000***
Indirect effect	CP⇒KT⇒SIP	0.5426	0.0794	0.3770	0.6931	0.000***
Total effect	CP⇒SIP	0.9207	0.0301	0.8615	0.9800	0.000***

CP = customer participation; KT = knowledge transfer; SIP = service innovation performance. LLCI refers to the lower limit of the estimated value 95% interval, and ULCI refers to the upper limit of the estimated value 95% interval. *** < 0.001.

## Conclusion

This study focuses on the analysis of the mechanism and boundary conditions of customer participation in virtual communities that affect enterprises’ service innovation performance, and specifically analyzes the relationship between the three dimensions of customer participation and the two dimensions of enterprises’ service innovation performance using knowledge transfer as an mediating variable. Through theoretical and empirical research methods, the hypotheses and model construction are verified, and there are several conclusion.

(1) Customer participation has a significant positive impact on the enterprises’ service innovation performance. This research establishes a research model of the relationship between customer participation and service innovation performance, and uses regression analysis and process methods to test the relationship between customer participation and innovation performance. Research shows that customer participation is beneficial to improving the enterprises’ service innovation performance. Customer participation helps enterprises obtain relevant knowledge such as customer needs and reduce barriers to knowledge sharing. In addition, enterprises can acquire customer knowledge about new products and help them successfully develop new products and services, thereby ultimately improving their service innovation performance.
(2) Knowledge transfer plays a mediating role between customer participation and service innovation performance. The results of this study support that knowledge transfer can mediate the positive relationship between customer participation and service innovation performance, indicating that the impact of customer participation on service innovation performance does not occur directly, but requires knowledge transfer to have an impact on service innovation performance. Knowledge transfer plays a mediating role between customer participation and service innovation performance, and also plays a part of the mediating role. From a logical point of view, when customers participate in the enterprise, they mainly participate in it by means of knowledge transfer, and thereby help the enterprise to improve service innovation performance. This conclusion shows that in the process of enterprises’ service innovation, customer participation will enhance the transfer of customer knowledge, which is conducive to the interaction between customers and enterprises. Enterprises carry out service innovation activities through the acquisition and application of customer knowledge, thereby enhancing their service innovation performance.

### Implications

The results of this study provide important implications.

(1) Enterprises must establish a comprehensive data system of customer behavior to guide customers to participate in depth. Customers in virtual communities have generated a lot of fragmented knowledge, but limited to the virtual nature of the community, customers cannot communicate face-to-face, and can only understand their expertise and communicate by capturing the behavioral dynamics of other customers. Through the investigation of virtual communities in multiple fields, it is found that the establishment of customer behavior data system in some communities is not very complete, which makes the willingness of customers to understand each other very low, which in turn leads to low customer activity in virtual communities. This research believes that the richness of customer behavior data display should be enhanced without infringing on user privacy, such as customer’s historical browsing, favorites, likes, publications and other behavioral dynamics, customer status data such as points, rank status, etc. As well as the customer’s skill tags and other data, it is important to establish a speciality catalog for customers who have just entered the virtual community, which is very important for the tendency to generate in-depth participation. It is necessary to promote the information sharing of customer participation in virtual community, and guide customers to update their expertise and share information through official recommendations and other methods to reduce cognitive duplication and information redundancy. It is necessary to promote the cooperative behavior of customer participation in virtual communities, strengthen communication between customers, and encourage customers to coordinate and cooperate to complete tasks. Enterprises or platforms should regularly initiate activities to guide customers to participate in mutual communication, understand the knowledge that customers have each other through communication, and build trust, fully integrate and use the information held by other customers, and improve the efficiency of task completion. It is necessary to promote the improvement of the interpersonal interaction ability of customer participation in virtual community. Enterprises or platforms should increase the flexibility, integration and interest of interactive channels by increasing the channels for customers to reach virtual communities. At the same time, optimize the interpersonal interaction process to enhance the customer’s experience of using the virtual community and create an immersive experience for customers. For example, design different development participation nodes and novel interactive methods to increase the participation of most silent customers, make the interactive memory system of the virtual community work better, and improve the service innovation performance.
(2) Enterprises should pay attention to customer knowledge management and improve the ability of customer

knowledge to be transformed into enterprises’ service innovation resources. Customer knowledge will help enterprises gain a deeper understanding of market positioning and more effectively grasp changes in market demand, thereby reducing the time and cost invested by enterprises in service innovation activities. The enterprise effectively integrates and applies the collected customer knowledge, so as to maximize the use of customer knowledge to optimize the original service and the research and development of new products, which in turn makes the service innovation performance of the enterprise continue to increase. At present, many enterprises are only passively collecting customer knowledge in virtual communities, or the customer knowledge has not been used rationally, and they have not been able to maximize the effect of customer knowledge in virtual communities. From the perspective of customer knowledge management in virtual communities, this is actually a waste of resources. Therefore, enterprises must actively collect customer knowledge in virtual communities, strengthen the management of customer knowledge, enhance the ability of customer knowledge to transform into enterprises’ service innovation resources, and ultimately improve service innovation performance.

### Limitations

This research still has certain limitations. Future research will be considered from the following aspects. First, this paper does not further explore the impact of different customer types in virtual communities on the enterprises’ service innovation performance. Due to the wide range of participants in virtual communities, the public groups for services will have different types due to factors such as customers’ participation motivation, customers’ personal abilities, and customers’ wishes. Studies have pointed out that different types of customers will affect the output quality of an organization according to their level of preference and resources they have (Rodie and Kleine, 2000; Claycomb et al., 2001). These different customer type factors may have an impact on the enterprises’ service innovation performance. Second, this paper does not explore the moderating factors of customer participation affecting service innovation performance. These moderating variables may have customer factors or enterprise factors. Customer’s social relationship quality, community network ability, participation attitude, task-related affective well-being, customer education are also important moderator variables that affect the relationship between customer participation and service innovation. Third, there are many types of virtual communities on the Internet at present. This study only explores the general attributes of virtual communities. The influence of customer participation on service innovation in different types of virtual communities and the particularity of the mechanism of action need to be explored in the future. In addition, the theoretical and empirical framework constructed in this paper will be extended to further study the antecedent variables of customer participation, explore the motivation of customer participation in service innovation, and find out which factors affect customer participation in service innovation behavior. For example, based on factors such as the characteristics, atmosphere, culture, and communication mechanism of the virtual community, it will have an impact on the motivation and degree of customer participation. Drawing on the theory of compensatory customer behavior (Wang et al., 2020, 2022), combined with the characteristics of customers, future research can also explore the impact of customer participation on service innovation from the perspective of service recovery or compensatory trust.

## Data Availability Statement

The original contributions presented in the study are included in the article/supplementary material, further inquiries can be directed to the corresponding author.

## Ethics Statement

The studies involving human participants were reviewed and approved by Ethics Committee of Changshu Institute of Technology. The patients/participants provided their written informed consent to participate in this study.

## Author Contributions

JW distributed the work done in the project, searched the background materials, designed the analytical characterization and empirical study frame, and has done the critical revision and editing.

## Conflict of Interest

The author declares that the research was conducted in the absence of any commercial or financial relationships that could be construed as a potential conflict of interest.

## Publisher’s Note

All claims expressed in this article are solely those of the authors and do not necessarily represent those of their affiliated organizations, or those of the publisher, the editors and the reviewers. Any product that may be evaluated in this article, or claim that may be made by its manufacturer, is not guaranteed or endorsed by the publisher.
